# Supplementary material for: Comparative Proteomics Reveals the Difference in Root Cold Resistance between Vitis. riparia × V. labrusca and Cabernet Sauvignon in Response to Freezing Temperature
Source: Plants (Basel). 2022 Apr 2;11(7):971. doi: 10.3390/plants11070971 (PMC9003149; doi:10.3390/plants11070971)
Supplement: Supplementary file 1 [file plants-11-00971-s001.zip › plants-1622859-supplementary.pdf]

Figure supplemental legends

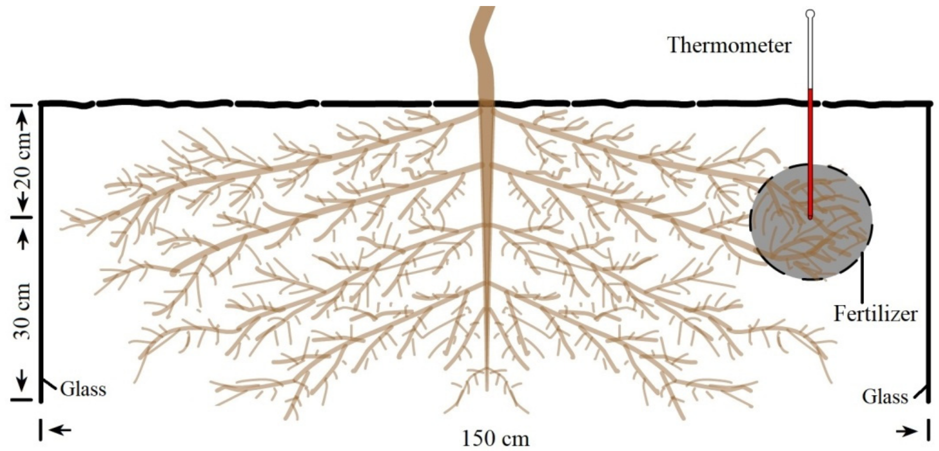

Figure S1. The model image of glass placed to separate the roots.

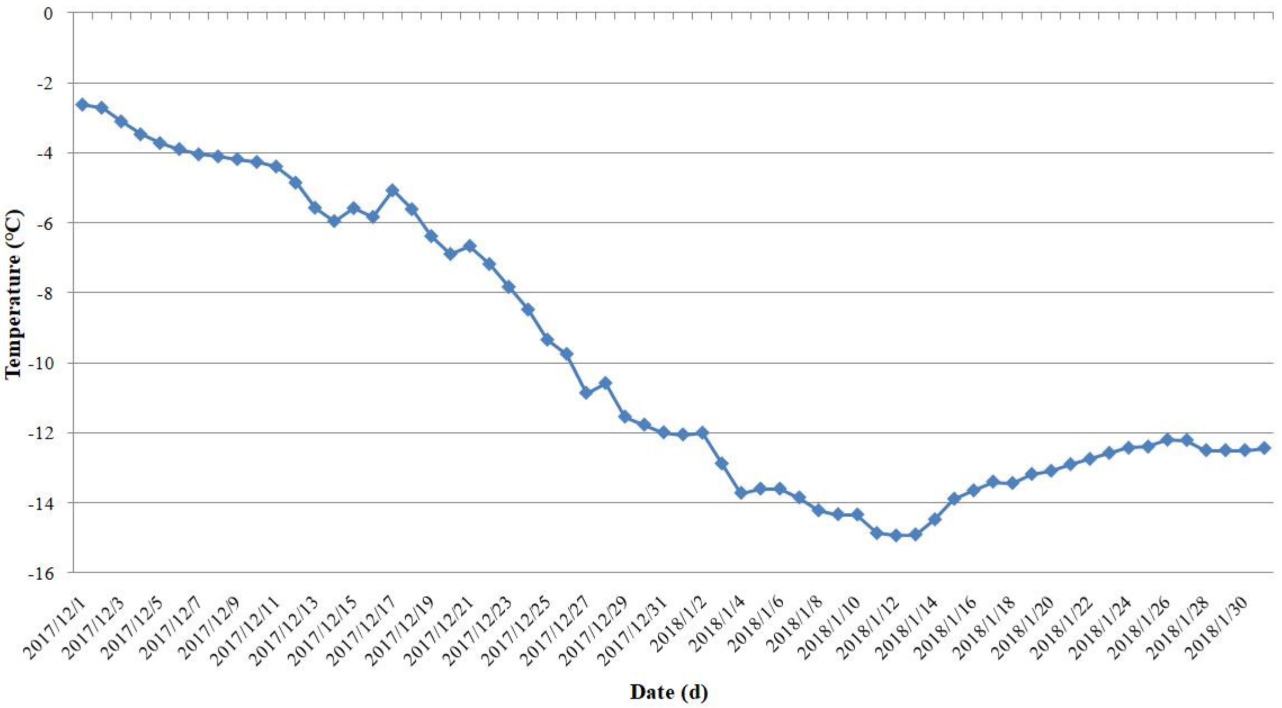

Figure S2. Changes of average temperatures at the 20 cm depth soil in December and January at the experiment site.

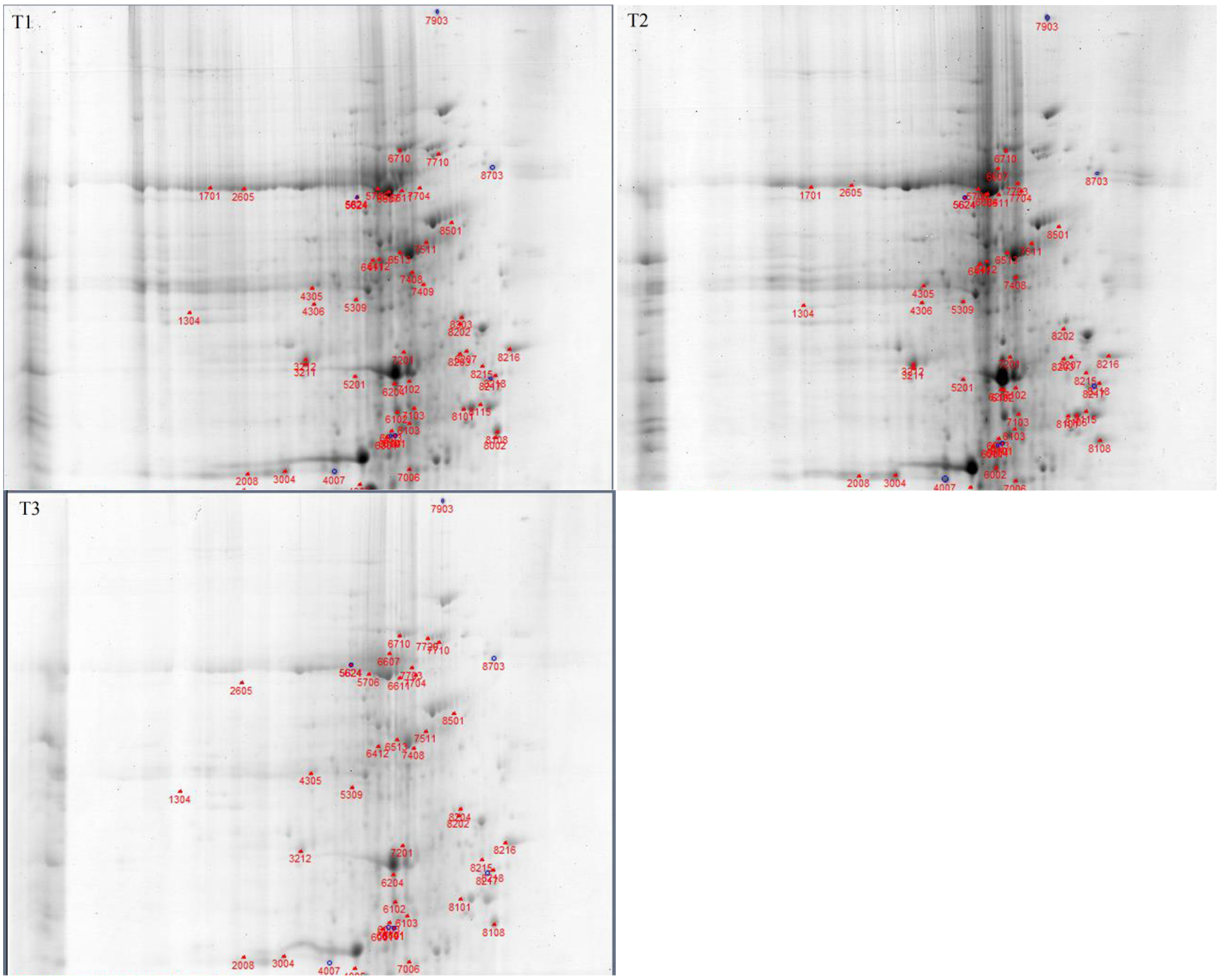

Figure S3. The representative images of SDS-PAGE at different treatments. Herein, T1: V. riparia × V. labrusca at -4°C; T2: V. riparia × V. labrusca at -15°C; and T3: Cabernet Sauvignon at -4°C.

Table supplemental legends

Table S1.Thirty-six DEPs identified in T2 vs. T1.

**Protein Name**	**SwissProt ID**	**log_2FC (T2 vs T1)**
**Stress response (6)**		
Dehydrin (DHN1)	Q4VT48	1.34
SHSP domain-containing protein (SHSPCP)	F6HJZ4	1.76
Usp domain-containing protein (USPCP)	F6H727	1.60
Ferritin (FER)	A5BV73	2.83
Glutaredoxin-dependent peroxiredoxin (GluDP)	A5ARL2	2.72
Glutathione peroxidase (GPX)	D7TW03	1.28
**Bio-signaling (4)**		
Protein kinase domain-containing protein (PKCP)	A5ALY7	1.44
Serine/threonine-protein phosphatase (S/TPP)	D7TV73	1.98
Non-specific serine/threonine protein kinase (nsS/TPK)	F6H1V3	1.75
Ubiquitin receptor RAD23 (RAD23)	D7T959	1.22
**Metabolism (7)**		
Alpha-1,4 glucan phosphorylase (GluP)	D7SXJ4	2.65
1,4-alpha-glucan branching enzyme (GluBE)	E0CQR2	0.58
Pectinesterase (PE)	F6HZ64	0.63
Abhydrolase 3 domain-containing protein (ABHD3CP)	F6HQC6	0.84
Proline iminopeptidase (ProIP)	D7T3J3	2.28
Methyltransferase (MT)	A5B620	2.61
Ubiquitin carboxyl-terminal hydrolase	F6H2F7	-1.63
**Energy (6)**		
ADP/ATP carrier protein (AAC)	A5BVR2	1.63
AAA domain-containing protein (AAACP)	D7TZI9	1.61
NAD(P)-bd dom domain-containing protein (NADCP)	F6HL96	1.59
NADH dehydrogenase [ubiquinone] 1 beta subcomplex subunit 7 (NDUFB7)	A5BAM7	1.82
Phytocyanin domain-containing protein (PCP)	A5C3C3	1.85
Succinate dehydrogenase [ubiquinone] flavoprotein subunit (SDHFS)	A5BGN3	1.69
**Translation (3)**		
Ribosomal protein L14 (rpL14)	B6VJZ7	2.61
40S ribosomal protein S21 (rpS21)	A5BUA6	2.76
Peptidylprolyl isomerase (PPI)	D7UDY0	1.61
**Transport (1)**		
Tubulin alpha chain	A5BZF5	-1.60
**Uncharacterized protein (9)**		
Uncharacterized protein	F6HSH9	-1.65
Uncharacterized protein	F6HNS2	-1.97
Uncharacterized protein	F6GV46	-2.74
Uncharacterized protein	D7TA50	-1.76
Uncharacterized protein	D7TVM3	-2.67
Uncharacterized protein	D7T1V9	1.42
Uncharacterized protein	F6HUZ8	1.88
Uncharacterized protein	F6HUA8	1.60
Uncharacterized protein	A5AQ98	-1.59

Table S2. Fifty-seven DEPs identified in T1 vs.T3.

Protein Name	SwissProt ID	log_2FC (T1 vs T3)
**Stress response (10)**		
Dehydrin (DHN1)	Q4VT48	1.78
SHSP domain-containing protein (SHSPCP)	F6HJZ4	2.01
Usp domain-containing protein (USPCP)	F6H727	2.69
Ferritin (FER)	A5BV73	1.83
Glutaredoxin-dependent peroxiredoxin (GluDP)	A5ARL2	1.92
Glutathione peroxidase (GPX)	D7TW03	1.73
Pathogenesis-related protein 10.3	B7SL50	-1.03
Peroxidase	D7SJL8	-1.14
PKS_ER domain-containing protein	D7TVV9	1.08
Stress-response A/B barrel domain-containing protein	A5C6X0	-1.06
**Bio-signaling (5)**		
Protein kinase domain-containing protein (PKCP)	A5ALY7	1.27
Serine/threonine-protein phosphatase (S/TPP)	D7TV73	2.92
Non-specific serine/threonine protein kinase (nsS/TPK)	F6H1V3	2.07
Ubiquitin receptor RAD23 (RAD23)	D7T959	1.35
Bet_v_1 domain-containing protein	A5C110	1.05
**Metabolism (14)**		
Alpha-1,4 glucan phosphorylase (GluP)	D7SXJ4	1.36
1,4-alpha-glucan branching enzyme (GluBE)	E0CQR2	1.57
Pectinesterase (PE)	F6HZ64	2.92
Abhydrolase 3 domain-containing protein (ABHD3CP)	F6HQC6	1.21
Proline iminopeptidase (ProIP)	D7T3J3	1.94
Methyltransferase (MT)	A5B620	1.75
AAI domain-containing protein	D7SLG6	-1.31
Aldo_ket_red domain-containing protein	D7TUK1	1.44
Fn3_like domain-containing protein	F6H0V8	1.45
Inositol-3-phosphate synthase	D7SVZ9	1.09
PCI domain-containing protein	D7TVK9	1.23
PfkB domain-containing protein	A5B8T3	1.19
Dirigent protein	A5B702	-1.44
Isoflavone reductase-like protein 5	Q3KN68	1.16
**Energy (7)**		
ADP/ATP carrier protein (AAC)	A5BVR2	1.61
AAA domain-containing protein (AAACP)	D7TZI9	1.59
NAD(P)-bd dom domain-containing protein (NADCP)	F6HL96	2.06
NADH dehydrogenase [ubiquinone] 1 beta subcomplex subunit 7 (NDUFB7)	A5BAM7	2.61
Phytocyanin domain-containing protein (PCP)	A5C3C3	2.83
Succinate dehydrogenase [ubiquinone] flavoprotein subunit (SDHFS)	A5BGN3	1.88
Complex1_30kDa domain-containing protein	A5AED5	1.66
**Translation (5)**		
Ribosomal protein L14 (rpL14)	B6VJZ7	2.80
40S ribosomal protein S21 (rpS21)	A5BUA6	1.67
Peptidylprolyl isomerase (PPI)	D7UDY0	2.51
Caffeoyl-CoA O-methyltransferase	A2TDB3	-1.65
M16C_associated domain-containing protein	F6HQC5	-2.80

**Transcription (1)**		
Integrase catalytic domain-containing protein	A5BVQ3	1.01
**Transport (2)**		
Cytochrome b5 heme-binding domain-containing protein	A5C997	-1.14
HMA domain-containing protein	F6HV23	-1.07
**Uncharacterized (13)**		
CAAD domain-containing protein	A5AEL3	-1.62
NmrA domain-containing protein	E0CQE7	1.69
VOC domain-containing protein	A5B1M7	-1.60
14_3_3 domain-containing protein	F6H2P0	-1.67
X8 domain-containing protein	D7SI17	-1.66
Uncharacterized protein	A5BL97	-1.49
Uncharacterized protein	A5AT21	-1.26
Uncharacterized protein	D7U7W7	1.04
Uncharacterized protein	F6H471	-1.58
Uncharacterized protein	F6HJM9	1.48
Uncharacterized protein	D7TYU4	-1.21
Uncharacterized protein	D7TZW2	-1.23
Uncharacterized protein	A5B6D2	-1.17
